# Pleiotropy constrains the evolution of protein but not regulatory sequences in a transcription regulatory network influencing complex social behaviors

**DOI:** 10.3389/fgene.2014.00431

**Published:** 2014-12-23

**Authors:** Daria Molodtsova, Brock A. Harpur, Clement F. Kent, Kajendra Seevananthan, Amro Zayed

**Affiliations:** ^1^Department of Biology, York UniversityToronto, ON, Canada; ^2^Department of Computer Science and Engineering, York UniversityToronto, ON, Canada

**Keywords:** *Apis mellifera*, network hubs, natural selection, evo devo, social evolution

## Abstract

It is increasingly apparent that genes and networks that influence complex behavior are evolutionary conserved, which is paradoxical considering that behavior is labile over evolutionary timescales. How does adaptive change in behavior arise if behavior is controlled by conserved, pleiotropic, and likely evolutionary constrained genes? Pleiotropy and connectedness are known to constrain the general rate of protein evolution, prompting some to suggest that the evolution of complex traits, including behavior, is fuelled by regulatory sequence evolution. However, we seldom have data on the strength of selection on mutations in coding and regulatory sequences, and this hinders our ability to study how pleiotropy influences coding and regulatory sequence evolution. Here we use population genomics to estimate the strength of selection on coding and regulatory mutations for a transcriptional regulatory network that influences complex behavior of honey bees. We found that replacement mutations in highly connected transcription factors and target genes experience significantly stronger negative selection relative to weakly connected transcription factors and targets. Adaptively evolving proteins were significantly more likely to reside at the periphery of the regulatory network, while proteins with signs of negative selection were near the core of the network. Interestingly, connectedness and network structure had minimal influence on the strength of selection on putative regulatory sequences for both transcription factors and their targets. Our study indicates that adaptive evolution of complex behavior can arise because of positive selection on protein-coding mutations in peripheral genes, and on regulatory sequence mutations in both transcription factors and their targets throughout the network.

## Introduction

Understanding the genetics and evolution of complex traits is a central goal in biology. Behavior is a complex phenotype that exhibits a high degree of variation within an individual's lifetime, within and between populations of the same species, and between species. Behavioral genetics research conducted over the past decade has emphasized the role of conserved genes in behavioral evolution. There is good evidence that behavior, like most complex phenotypes, is controlled by gene regulatory networks that exhibit modularity and pleiotropy, and that genes and gene networks that influence behavior in one organism also influence similar behaviors in evolutionary distant species (Anholt and Mackay, 2004; Reaume and Sokolowski, 2011; Zayed and Robinson, 2012). This conservation of gene action on behavior has allowed researchers to study behavioral evolution within the framework of Evolutionary Developmental Biology (i.e., evo devo) (Carroll, 2008). The synthesis of behavioral genetics and evo devo has led to many insights (Linksvayer and Wade, 2005; Toth and Robinson, 2007, 2009), including the existence of a genetic tool kit for behavior (i.e., conserved gene modules that influence basic forms of behavior across species), and that complex behaviors can evolve through the co-option of genetic modules that control simple forms of behavior. In contrast to the evo devo paradigm, there is a burgeoning body of literature suggesting that novel taxonomically-restricted genes are important, and perhaps most prominent, in behavioral evolution (Johnson and Tsutsui, 2011; Chen et al., 2013; Ferreira et al., 2013; Simola et al., 2013; Harpur et al., 2014; Jasper et al., 2014; Sumner, 2014). Fortunately, genomics-enabled research on a variety of model and non-model organisms is providing a wealth of information on the contribution of novel and conserved genes to the genetic architecture of complex traits. Along with population genomic data on levels of selection acting on genes and regulatory sequences, evolutionary biologists are at the verge of ultimately testing the different theories of phenotypic evolution.

The different paradigms of phenotypic evolution make distinct predictions about the relative contribution of regulatory and protein-coding sequence changes. On one end of the spectrum, the evo devo paradigm emphasizes the role of adaptive regulatory sequence evolution (Wray, 2007; Carroll, 2008) because of the assumption that genes with multiple functions, or genes that interact with other genes, are expected to experience a great deal of constraint at their amino acid sequence (Fisher, 1930). Others have challenged this central assumption of the evo devo paradigm by arguing that seemingly “conserved” proteins, including transcription factors, have several features that allow them to “escape” the constraining influence of pleiotropy thereby allowing adaptive evolution via amino-acid changing mutations (Lynch and Wagner, 2008; Wagner and Lynch, 2008); such features include alternative splicing, modularity at the level of protein domain and structure, and the presence of mutable short or simple sequence motifs. At the other end of the spectrum, there is a growing interest in novel taxonomically restricted genes that are free to evolve new functions without suffering from the constraining effect of pleiotropy (Chen et al., 2013). Empirical evidence do not fully support any one of these three paradigms over the others—there is population genetic evidence for both adaptive protein sequence evolution and adaptive coding sequence evolution in many organisms (Andolfatto, 2005; Hoekstra and Coyne, 2007; Halligan et al., 2010, 2013; Harpur et al., 2014; Wallberg et al., 2014). However, most previous tests of these paradigms involved correlating general rates of protein evolution with molecular features of genes and their position in regulatory networks (e.g., Hahn and Kern, 2005; Kim et al., 2007; Davila-Velderrain et al., 2014); data on the actual levels of positive or negative selection on coding sequences (Assis and Kondrashov, 2014) are seldom used. Moreover, we know very virtually nothing about how pleiotropy and the structure of gene regulatory networks affect patterns of regulatory sequence evolution.

The honey bee *Apis mellifera* has emerged as a model organism for studying the genetics and evolution of complex behaviors (Hunt et al., 2007; Page et al., 2012; Zayed and Robinson, 2012). Here we use several powerful genomic resources developed for the honey bee to examine if regulatory networks that influence behavior follow the predictions of the evo devo paradigm for phenotypic evolution. Chandrasekaran et al. (2011) recently constructed a brain transcriptional regulatory network (TRN) influencing several aspects of worker behavior, including behavioral maturation, foraging, and colony defense. The honey bee brain TRN is highly amenable to studies of how connectedness and network topology constrain behavioral and molecular evolution, especially given the recent availability of a large population genomic dataset for the honey bee (Harpur et al., 2014), which consists of genome wide polymorphism data for 39 *A. mellifera* diploid genomes and genome wide divergence data between *A. mellifera* and its sister species *A. cerana*.

We used the honey bee population genomic dataset to study the strength of selection on protein and putative *cis*-regulatory sequences of genes in the bee brain TRN. We tested the following hypotheses from the evo devo paradigm: (1) Highly connected TFs and target genes are predicted to experience stronger negative selection on nonsynoymous mutations relative to weakly connected TFs and target genes and (2) Genes with signs of adaptive amino acid sequence evolution are expected to be less central within the regulatory network. The evo devo paradigm does not explicitly make predictions about the relationship between pleiotropy and regulatory sequence evolution, but rather predicts that the evolution of regulatory sequences should be less constrained relative to protein sequence evolution, and that regulatory mutations are more likely to fuel adaptive evolution. We compared the average selection coefficient on mutations in putative *cis*-regulatory regions of strongly and weakly connected genes within the TRN to explore how network properties influence regulatory sequence evolution. Our study provides an important glimpse into the evolution of regulatory networks that influence complex behaviors.

## Materials and methods

### Sequencing, alignment, SNP calling and modified McDonald-Kreitman (MK) tests

We recently sequenced 40 honey bee genomes, each at approximately 40X coverage, using Illumina Hi-Seq technology (Harpur et al., 2014). Alignment and polymorphism identification were described in detail by Harpur et al. (2014). We used a Bayesian implementation of the McDonald-Kreitman (MK) test, using SnIPRE (Eilertson et al., 2012), to determine the population size scale selection coefficient γ for 12,303 genes in the honey bee genome. Here, we used the population genomics dataset to study selection acting on putative *cis*-regulatory regions of the honey bee genome. We first estimated the number of polymorphic mutations in *A. mellifera*, and the number of fixed mutations between *A. mellifera* and its sister species *A. cerana*, in putative *cis*-regulatory regions of honey bee genes. Because the regulatory sequences of the honey bee genome have not been characterized, we considered the 1000 bp sequence upstream of each gene's start codon as a putative *cis*-regulatory region (Davidson, 2006; Li et al., 2006; Myers, 2014). We excluded upstream sequences that overlapped genes encoded by the complementary DNA strand, resulting in putative *cis*-regulatory regions with an average size of 905 bp. These regions are expected to contain most of the sequences important for transcriptional and translational control, including the 5'UTR and important transcription factor binding sites (Davidson, 2006; Li et al., 2006; Myers, 2014). Our cut-off would have certainly excluded some regulatory sequences that reside far upstream of genes (Negre et al., 2011)—sequences that are currently very difficult to annotate in the honey bee. Despite this important caveat, our population genomic analyses (see results) show an overall signature of negative purifying selection within 1 Kb upstream of genes, which is consistent with such regions having a functional role related to gene regulation (Dunham et al., 2012; Wittkopp and Kalay, 2012). Following, Torgerson et al. (2009), we studied the evolution of *cis*-regulatory regions using a modified MK test by comparing the ratio of fixed:polymorphic mutations in a *cis*-regulatory sequence of a gene to same ratio for silent sites in the same gene. The modified MK test was implemented using SnIPRE (Eilertson et al., 2012), which allowed us to estimate the average population size scaled selection coefficients on regulatory sequence mutations. Similar to Harpur et al. (2014), we only used polymorphism data from African honey bee genomes, which represent a large population that is minimally impacted by human management (Harpur et al., 2012; Kent et al., 2012).

### TRN construction and analysis

The honey bee brain TRN (Chandrasekaran et al., 2011) is freely available online (Web: http://price.systemsbiology.net/honeybee-transcriptional-regulatory-network). The dataset consisted of microarray probes for TFs and their targets in the bee brain TRN. We remapped the array probes to the honey bee's official gene set OGS v3.2 (Elsik et al., 2014) using Blastn v. 2.2.28+. We only retained probes that had perfect matches to OGS v3.2 gene predictions. We were able to blast match microarray probes to 191 transcription factors and 1597 target genes. We restricted our analyses to 184 TFs and 1521 target genes that had γ estimates for coding and putative regulatory sequences. We estimated the number of target genes for every transcription factor (k ranged from 1 to 161), and the number of transcription factors regulating every target (k ranges from 1 to 15). We plotted the regulatory network using Gephi (Bastian et al., 2009) and produced a directed graph with 1504 nodes and 5149 edges representing transcription factor—target interactions. Gephi was used to estimate *betweenness* centrality of the genes in the network. We used the R package poweRlaw (Gillespie, 2014) to fit a power law distribution to TRN connectedness using established methods (Clauset et al., 2009). Statistical tests were carried out using R. We used a one-tailed test to compare the γ of hub and non-hub TFs and targets, given *a priori* theoretical expectations and empirical findings regarding the relationship between pleiotropy/connectedness and molecular evolution. All other *p*-values are two-tailed. It is important to note that the honey bee brain TRN was developed by first selecting honey bee TFs that had robust orthologs to *Drosophila* TFs (Chandrasekaran et al., 2011); the bee brain TRN is thereby enriched for old taxonomically-conserved TFs and target genes. Our study of the bee brain TRN can therefore illuminate how ancestral gene networks influencing behaviors evolve, but tell us little about the role of taxonomically-restricted genes in behavioral evolution—a topic that we recently discussed elsewhere (Harpur et al., 2014).

## Results

### Selection on regulatory and coding sequences in the honey bee genome

**Figure 1:**
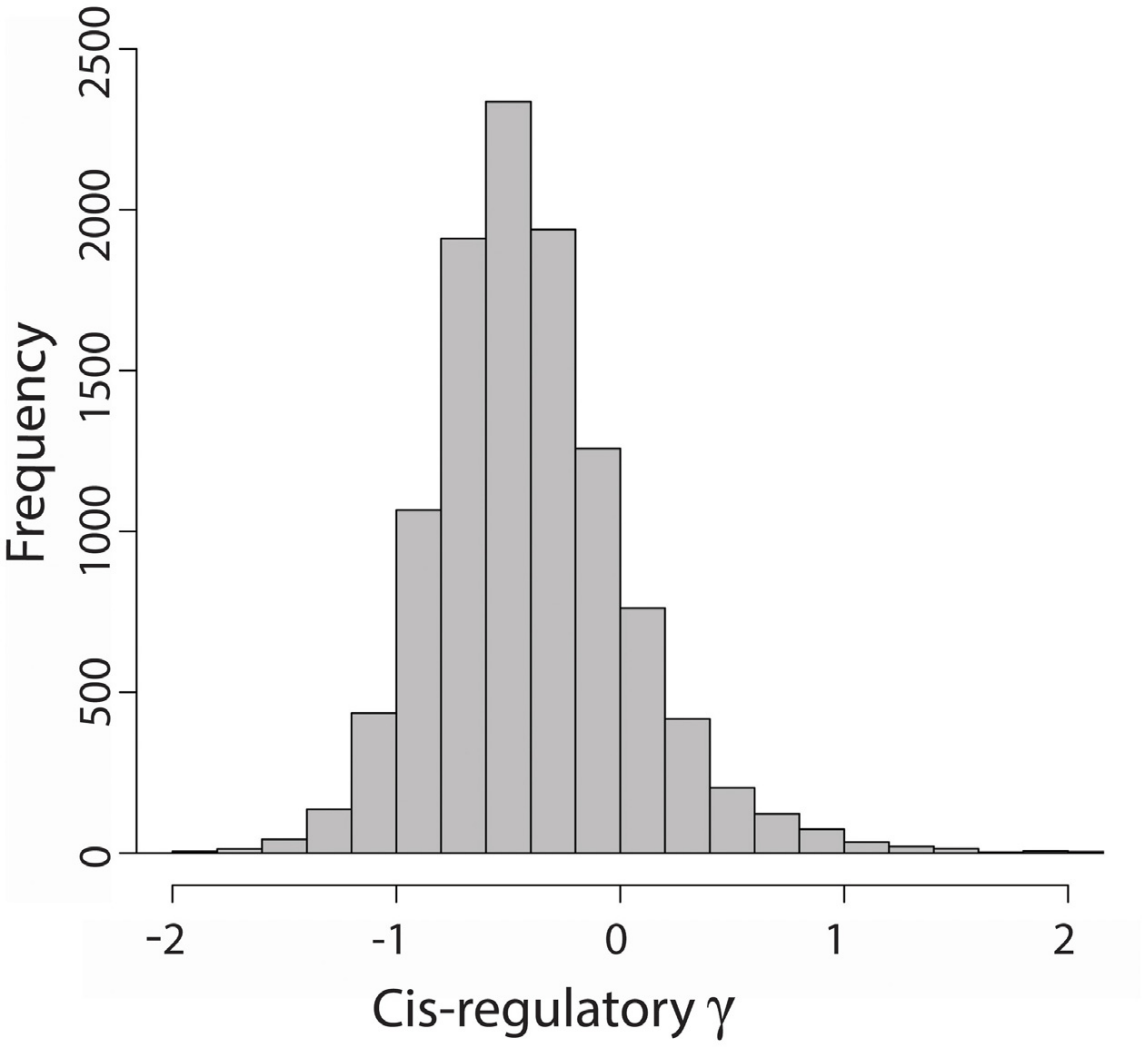
**Distribution of average population size scaled selection coefficients (γ) on *cis*-regulatory mutations in 10,807 genes in the honey bee genome**. Ten genes with *cis*-regulatory γ > 2 were omitted from the histogram for readability.

We had previously estimated the average population size scaled selection coefficient γ on nonsynonymous mutations in 12,303 genes in the honey bee genome since divergence between *A. mellifera* and *A. cerana* (ca. 5 MYA) (Harpur et al., 2014). Here we used a variant of the MK test (Torgerson et al., 2009; implemented using Eilertson et al., 2012) to estimate the average γ on mutations in putative *cis*-regulatory sequences by comparing the ratio of polymorphic:fixed mutations within 1 kb upstream of a gene's start codon to the ratio of polymorphic:fixed synonymous mutations at the same gene. We were able to estimate γ on the putative *cis*-regulatory sequences of 10,807 genes in the honey bee genome (Figure 1). We found most (93%) *cis*-regulatory sequences to have estimates of γ consistent with neutral or nearly neutral evolution (−1 < γ < 1). About 6% of *cis*-regulatory sequences have γ < −1, indicative of negative purifying selection, while 1% of sequences have signs of positive selection (γ > 1). In contrast to evolution of protein coding sequences (average γ ~ 0), the average mutation in *cis*-regulatory regions appear to be weakly deleterious (average γ = −0.4). This pattern was previously observed in humans (Torgerson et al., 2009) and most likely results from an observational bias: sequences from rapidly evolving regulatory regions will have many mismatches between *A. mellifera* and *A. cerana*, which results in lower alignment scores and coverage, and would have been removed from the dataset based on our quality control filters. As such, direct comparisons of the selection coefficient on coding and regulatory mutations are not appropriate. Instead, we examined the influence of a gene's connectedness and position within the TRN on regulatory and protein sequence evolution in separate analyses.

### Network topology and evolution of TFs and their target genes

**Figure 2:**
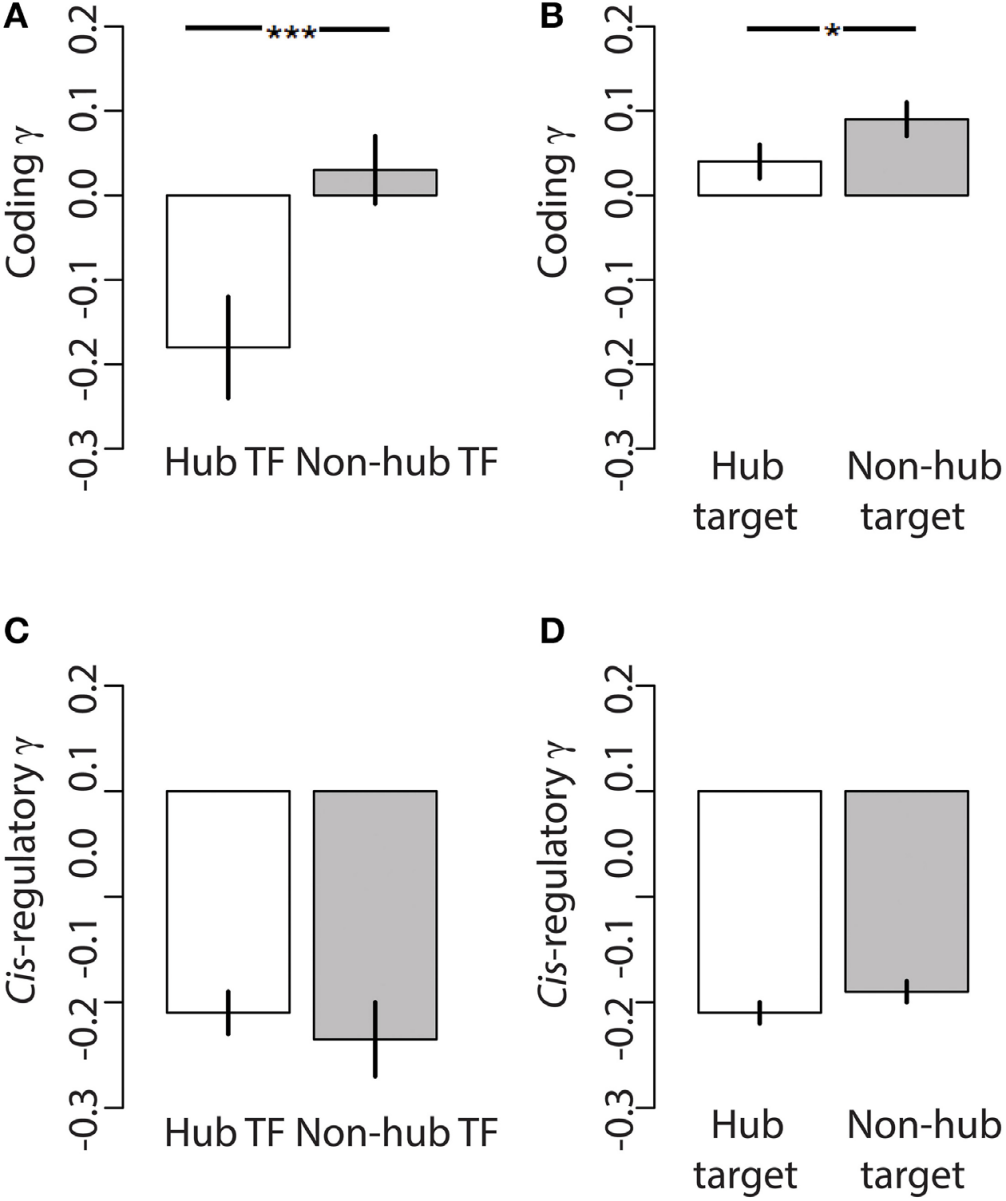
**Connectedness reduces the seletion coefficient on coding but not regulatory mutations across the honey bee TRN**. Both **(A)** hub TFs and **(B)** hub target genes have signficantly stronger negative selection on their coding sequences (i.e., lower coding γ) relative to non-hub TFs and non-hub targets, respectively. The selection coefficient on putative *cis*-regulatory sequences of **(C)** hub TFs and **(D)** hub target genes do significantly differ relative to non-hub TFs and non-hub targets, respectively. Bars indicate Mean ± SEM, ^*^p < 0.05, ^***^p < 0.001.

We studied patterns of selection on coding and regulatory mutations in 170 transcription factors (TFs) and 1334 of their target genes in the honey bee brain TRN. Similar to other regulatory networks (Babu et al., 2004; Nicolau and Schoenauer, 2009), the honey bee brain TRN is approximately scale-free, whereby the distribution of connectedness (*k*) between the network nodes (i.e., genes) has a very long tail (Supplementary Information Figure S1). The bee brain TRN contained a large number of genes with a small number of connections, and a small number of genes with a large number of connections—often called “hub” genes. The number of connections, *k*, between nodes in a scale-free network follows a power law, at least above a certain value of *k* (Nicolau and Schoenauer, 2009). Connectedness varied between 1 and 161 in the honey bee brain TRN, and we found the tail of the connectedness distribution to follow a power law (*x*_min_ = 42, ∝ = 3.00; *H*_0_ = power law: Goodness of fit: 0.088, *p* = 0.32). We elected to analyse the dataset by categorizing genes as hub or non-hub, following Wang et al. (2010a), because analyses based on linear models or correlations do not adequately deal with the properties of regulatory networks (i.e., the distribution of connections within the TRN is not normal). Following Wang et al. (2010a), we considered the top 20% of most connected TFs as hubs (*k* > 44 connections). Hub TFs were more central in the network as evidenced by a significantly higher estimate of eigenvector centrality relative to non-hub TFs (Wilcoxon test, *p* < 2.2e-16). We found that hub TFs had a significantly lower mean coding γ than non-hub transcription factors (Figure 2A, Wilcoxon 1-tailed *p* = 0.0025), and that hub TFs were significantly enriched for genes with negative coding γ (Chi square enrichment *p* = 0.015) relative to non-hub TFs. In contrast to coding γ, hub TFs and non-hub TFs did not significantly differ with respect to *cis*-regulatory γ (Figure 2C, Wilcoxon 1-tailed *p* = 0.27). Hub and non-hub TFs did not significantly differ in terms of sequence coverage and length at regulatory and coding sites (Supplementary Information Table 1).

Similar to TFs, we used connectedness to classify target genes in the TRN into hubs (top 20%) and non-hubs based on *k*. Hub target genes within the TRN were regulated by four or more TFs, and were significantly more central within the network relative to non-hub target genes (Wilcoxon *p* = 2.2e-16). Similar to the differences between hub TFs and non-hub TFs, hub target genes had significantly lower coding γ (Figure 2B, Wilcoxon 1-tailed *p* = 0.0425), but not *cis*-regulatory γ (Figure 2D, Wilcoxon 1-tailed *p* = 0.12) relative to non-hub target genes. Hub and non-hub target genes did not significantly differ in terms of sequence coverage and length at regulatory and coding sites (Supplementary Information Table 1).

### Where is positive selection acting within the TRN?

**Figure 3:**
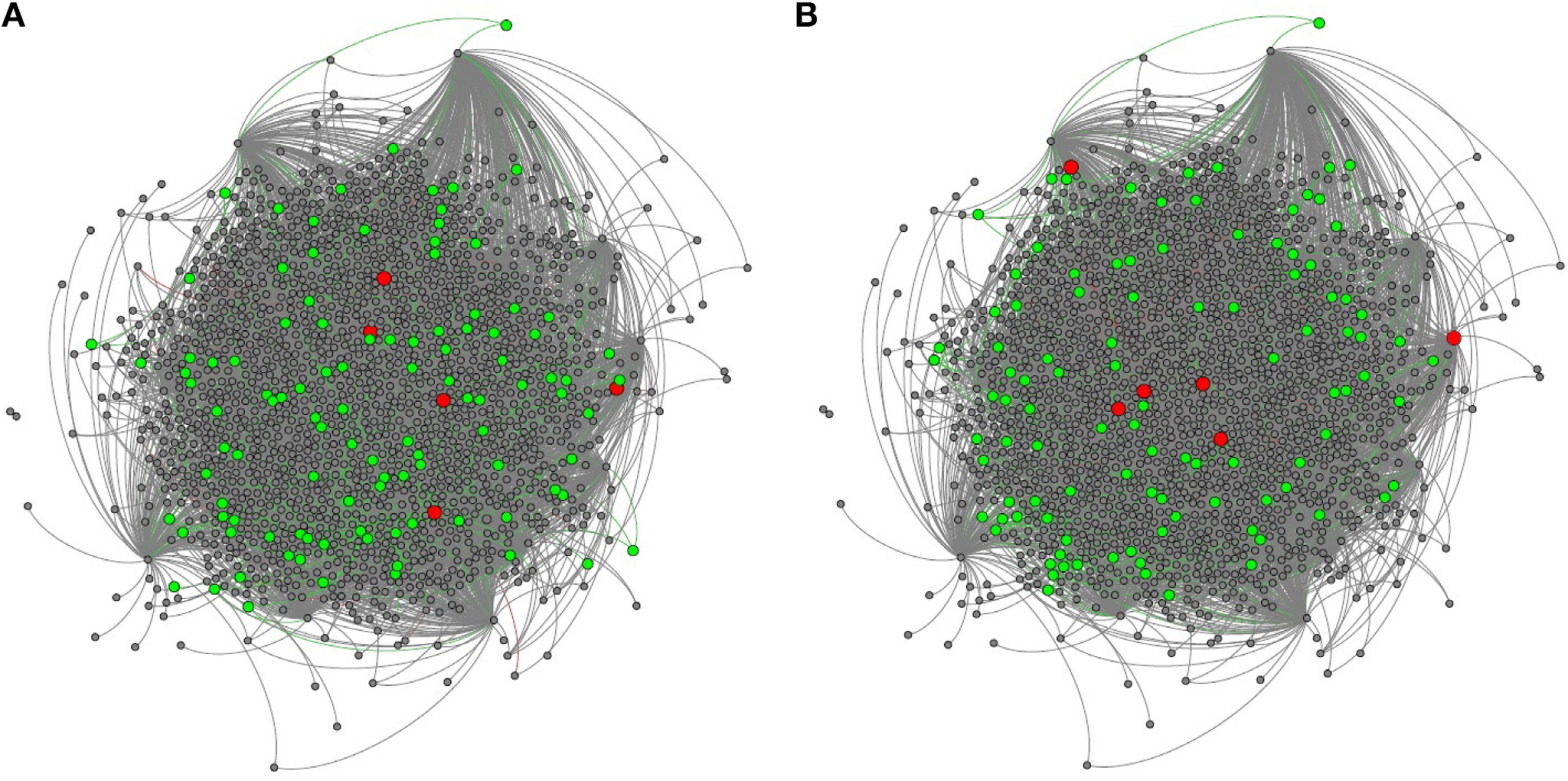
**The honey bee brain TRN highlighting genes with adaptively evolving (A) *cis*-regulatory and (B) coding sequences**. Adaptively evolving transcription factors are highlighted in red, while adaptively evolving targets are highlighted in green.

**Figure 4:**
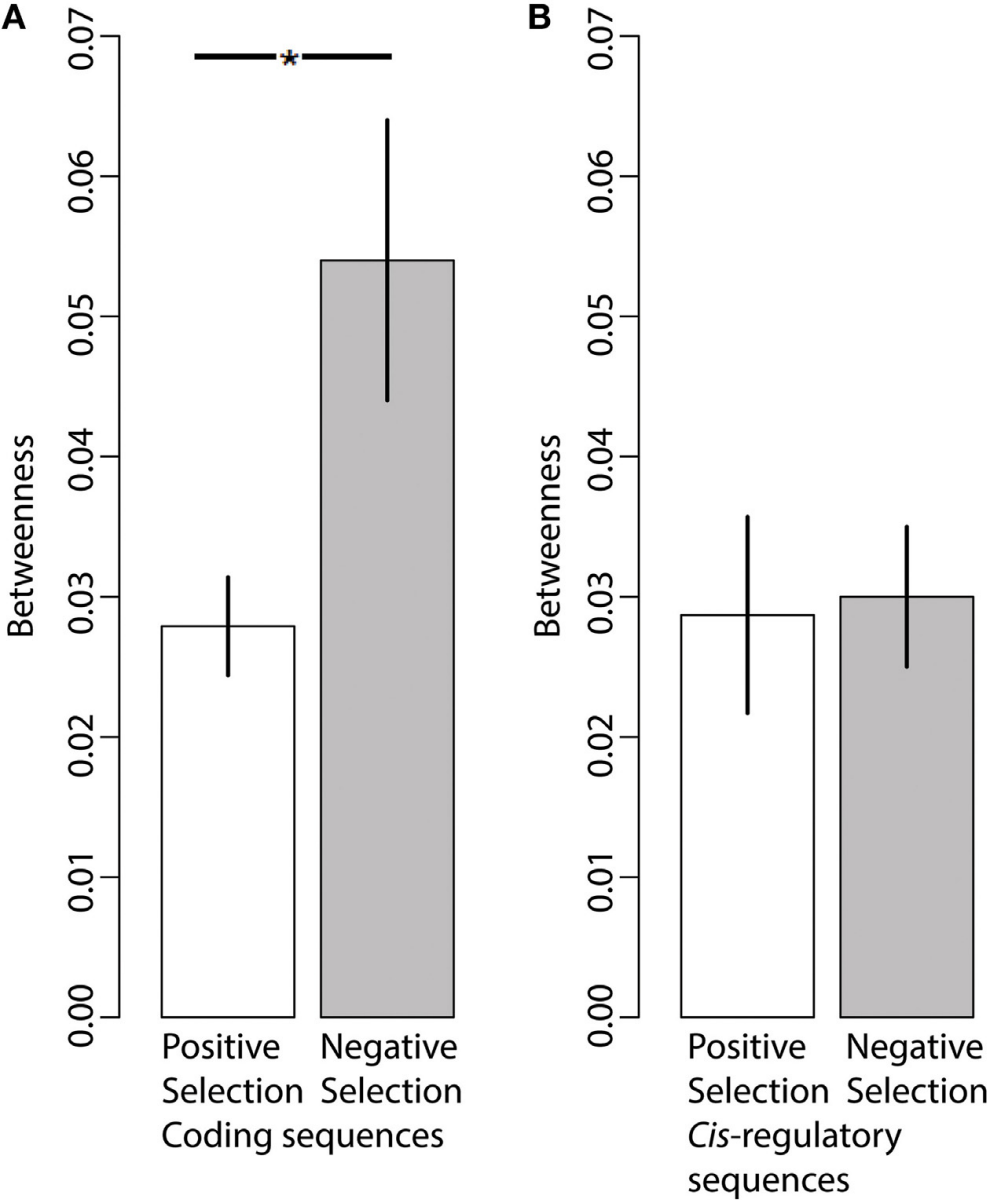
**Network position is associated with differences in coding sequence evolution but not regulatory sequence evolution**. **(A)** Genes experiencing positive selection (γ > 1)on their coding sequences (*N* = 105) have significantly lower *Betweenness* centrality estimates (i.e., are further away from the network core) relative to genes experiencing negative selection (γ < −1) on their coding sequences (*N* = 7). **(B)** The average *Betweenness* centrality of genes experiencing positive selection (γ > 1) on their regulatory sequences (*N* = 16) does not significantly differ relative to that of genes experiencing negative selection (γ < −1) on their regulatory sequences (*N* = 92). Bars indicate Mean ± SEM. ^*^p < 0.05.

We mapped all genes with signatures of positive selection on coding and *cis*-regulatory sequences in the TRN (Figure 3). We also estimated *betweenness* for each gene in the TRN; *betweenness* is a global measure of centrality (Borgatti and Everett, 2006) which ranges from 1, indicating most central or at the core of the network, to 0, indicating the outside perimeter or the periphery of the network. We compared the average *betweenness* of genes with substantial signs of positive (γ > 1) and negative (γ < −1) selection. We found that proteins with signatures of positive selection on their coding sequences had significantly lower *betweenness* relative to proteins with signatures of negative selection, indicating that adaptively evolving proteins are often more distant from the network core relative to proteins with signs of negative selection (Figure 4A, Wilcoxon, two tailed *p* = 0.04). In contrast, we did not find a significant difference in the *betweenness* of genes with positive selection on their *cis*-regulatory sequences relative to those with negative selection on their *cis*-regulatory sequences (Figure 4B, Wilcoxon two-tailed *p* = 0.4). This indicates that genes with regulatory sequences experiencing positive selection reside in approximately the same locations within the TRN as genes with regulatory sequences experiencing negative selection.

## Discussion

We examined how gene position within a network influenced the average selection coefficient γ on putative *cis*-regulatory and replacement mutations in 1504 genes in the honey bee brain TRN. Our results support a “mosaic” view of phenotypic evolution by illuminating how the scale-free properties of regulatory networks (Wang et al., 2010b; Le Nagard et al., 2011; Wagner and Zhang, 2011) facilitate adaptive evolution involving both coding and regulatory mutations.

Several lines of evidence suggest that the most connected, and likely most pleiotropic, proteins within the bee brain TRN experience the greatest levels of purifying selection, as predicted by Fisher (1930) and the Evo Devo paradigm (Carroll, 2008). Despite the large number of factors that influence the rate of molecular evolution of genes (Xia et al., 2009) we consistently found that the most connected genes in the TRN had the strongest signatures of negative selection on their coding sequence. In brief, transcription factors that regulate hundreds of target genes experience, on average, stronger negative selection on their coding sequence relative to transcription factors the regulate a few target genes (Figure 2A). Hub transcription factors likely have to interact with many other co-factors, in addition to binding target promoter sites, which may be responsible for the stronger levels of purifying selection on their amino acid sequence. Similar to hub transcription factors, hub target genes that are regulated by many transcription factors experience stronger negative selection on their coding sequence relative to target genes that are regulated by a few transcription factors. Target genes that are regulated by multiple TFs may be expressed in multiple tissues or during multiple contexts relative to target genes regulated by a few TFs, resulting in greater pleiotropy and stronger purifying selection, as evident from our analysis (Figure 2B). It is important to note that several genes within the TRN had signs of adaptive protein evolution; most of these genes were transcription factor targets, and most resided near the periphery of the TRN. Lynch and Wagner (2008) and Wagner and Lynch (2008) previously argued that proteins, including conserved TFs, have features that allow them to escape from the negative effects of pleiotropy. Our population genomic data are not fully consistent with the Lynch and Wagner hypotheses because the most central and most connected TFs or targets do experience stronger levels of negative selection vs. peripheral and weakly connected TFs or targets; a relationship that is more inline with the classic evo devo paradigm. We strongly believe that the structure of TRNs hold the key for reconciling the predictions of the evo devo paradigm with the empirical data showing that amino-acid changes do contribute to adaptive evolution. The classic evo devo paradigm assumes that *most* genes are constrained by pleiotropy, while studies of TRN structure clearly show that only a *few* genes are highly connected and central, while *most* genes are weakly connected and peripheral. Although pleiotropy does appear to curtail adaptive protein sequence evolution of the *few* most connected and most central genes within a TRN, adaptive protein evolution is still a powerful evolutionary force for *most* TRN genes that reside *at* the network periphery.

In stark contrast to the influence of TRN topology on protein coding evolution, we found that connectedness matters little with respect to levels of selection on putative *cis*-regulatory regions. The average selection coefficient on regulatory sequence mutations of hub transcription factors was similar to that of non-hub transcription factors (Figure 2C). Similarly, the selection coefficient on regulatory sequences of hub target genes was similar to those of non-hub target genes. Genes with signs of adaptive regulatory sequence evolution were found in similar locations within the TRN as genes with negative selection on their regulatory sequences. Our analysis indicates that network properties do not significantly shape the selection pressures acting on regulatory sequences within the TRN. It is not clear how this evidence support the evo devo paradigm because the evo devo paradigm does not make explicit predictions about the relationship between pleiotropy, connectedness and regulatory sequence evolution. On one hand, our finding that putative *cis*-regulatory sequences evolve independently of TRN connectedness and topology appears to support an important assumption of the evo devo paradigm: pleiotropy or connectedness of a protein only influences the protein's amino acid sequence, not its *cis*-regulatory sequence. On the other hand, another interpretation of the evo devo paradigm suggests that the most connected and pleiotropic genes should have the greatest levels of adaptive regulatory evolution, while the least connected genes should have the least levels of adaptive regulatory evolution (i.e., regulatory sequence evolution compensates for constrained amino acid sequences); our findings do not support this idea. It would appear that adaptive regulatory sequence evolution can occur throughout any compartment of the regulatory network.

Our analyses shed light on the evolution of regulatory networks influencing complex behavior. Highly connected genes within the honey bee brain TRN exhibit stronger patterns of purifying selection on amino acid replacement mutations similar to highly connected genes in other types of networks studied so far. Also, genes with signs of adaptive protein evolution tend to be concentrated at the network periphery, as previously documented for proteins in the Human Interactome (Kim et al., 2007). We found that connectedness does not influence the strength of selection on regulatory sequences of genes in the bee brain TRN. Our study suggests that the properties of regulatory networks, with a few large modules and many small modules, allows for both coding and regulatory sequence mutations to contribute to adaptive evolution. Based on our findings, we expect adaptive evolution of regulatory networks influencing complex traits to proceed through positive selection on coding mutations in peripheral genes and on regulatory mutations in TFs and their targets across the regulatory network. We had previously presented strong evidence that novel taxonomically-restricted genes have the highest rates of adaptive protein evolution in the honey bee genome (Harpur et al., 2014). A recent analysis also pointed to an increased expansion of regulatory sequences in social genomes (Simola et al., 2013). Going forward, it will be important to study how novel taxonomically restricted genes interact with conserved TRN modules with expanded regulatory features to influence the evolution of complex behaviors in social insects.

### Conflict of interest statement

The authors declare that the research was conducted in the absence of any commercial or financial relationships that could be construed as a potential conflict of interest.

## Supplementary material

Figure S1 **Degree distribution of connectedness in the TRN**.
