# Supplementary figures and images for: Pleiotropy constrains the evolution of protein but not regulatory sequences in a transcription regulatory network influencing complex social behaviors
Source: Front Genet. 2014 Dec 23;5:431. doi: 10.3389/fgene.2014.00431 (PMC4275039; doi:10.3389/fgene.2014.00431)

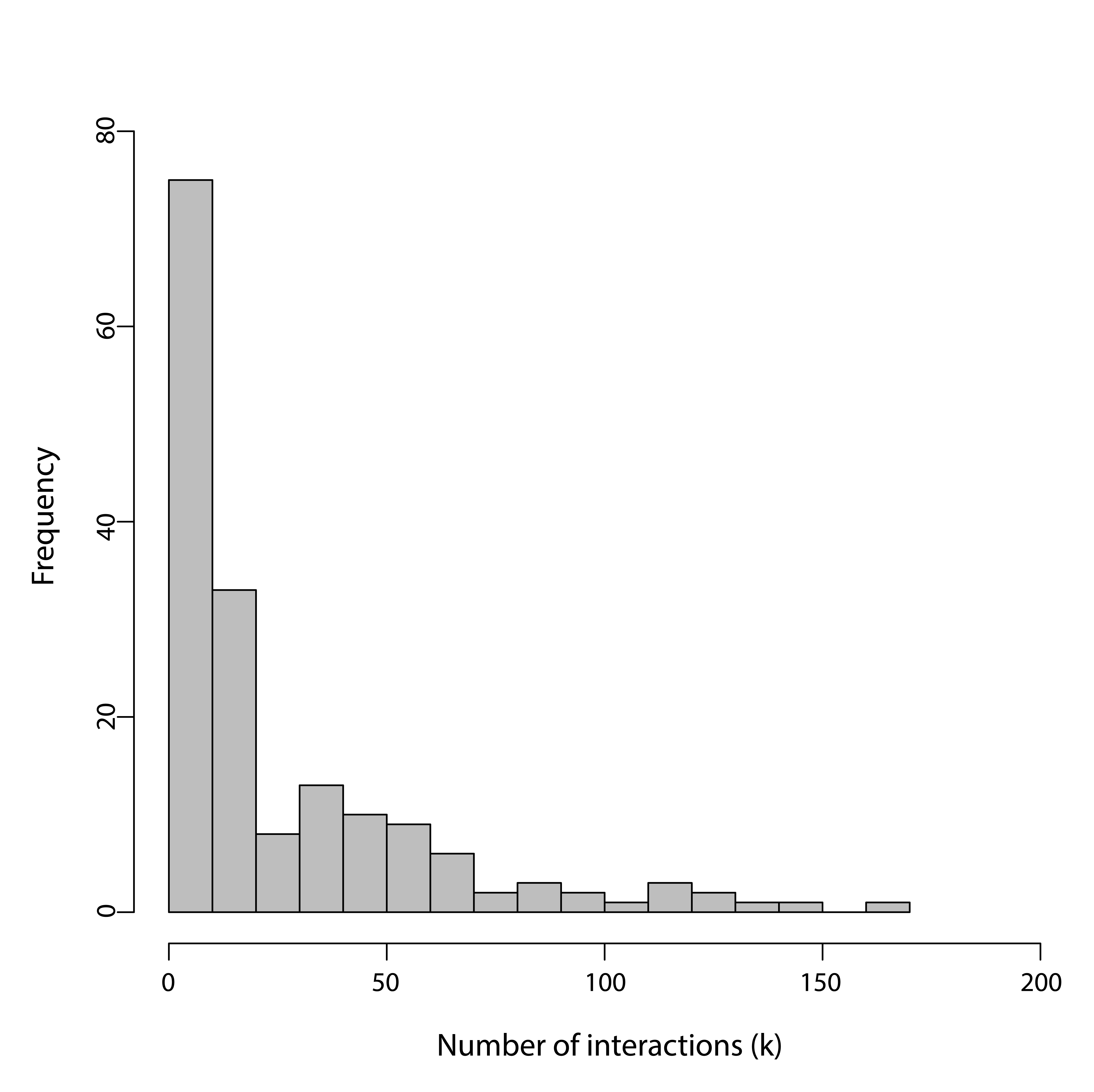

Supplement: Figure S1 — Degree distribution of connectedness in the TRN. [file Image1.JPEG]
