# Supplementary material for: Pleiotropy constrains the evolution of protein but not regulatory sequences in a transcription regulatory network influencing complex social behaviors
Source: Front Genet. 2014 Dec 23;5:431. doi: 10.3389/fgene.2014.00431 (PMC4275039; doi:10.3389/fgene.2014.00431)
Supplement: Supplementary file 2 [file Table1.DOCX]

**Pleiotropy constrains the evolution of protein but not regulatory sequences in a transcription regulatory network influencing complex social behaviours.**

Daria Molodtsova^1^, Brock A. Harpur^1^, Clement F. Kent^1,2^, Kajendra Seevananthan^1^, Amro Zayed^1,3^_._

^1^Department of Biology and ^2^ Department of Computer Science and Engineering, York University, Toronto, ON, Canada

^2^ Present address: HHMI Janelia Farm Research Campus, Ashburn, VA, USA

**Correspondence:**
Dr. Amro Zayed
Department of Biology
York University
4700 Keele Street, Toronto
M3J 1P3, ON, Canada
zayed@yorku.ca

**Short Title:** Evolution of bee regulatory network

**Table S1.** Coverage and sequence length for *cis*-regulatory and coding regions of the genes in the TRN. Wilcoxon rank sum test 2-tailed p-values are reported for all tests.

| Groups compared | Mean±SEM | p value |
| --- | --- | --- |
| Average cis-regulatory read depths of hub and non-hub TFs | hub=33.5±0.9  non-hub=34.6±0.5 | 0.5 |
| Average cis-regulatory read depths of hub and non-hub targets | hub=33.8±0.2  non-hub=33.9±0.2 | 0.4 |
| Average number of bps covered in cis-regulatory region of hub and non-hub TFs | hub=895±34.4  non-hub=855±19.5 | 0.2 |
| Average number of bps covered in cis-regulatory region of hub and non-hub targets | hub=798.5±12.8  non-hub=830.7±8.7 | 0.1 |
| Average coding read depths of hub and non-hub TFs | hub=37.4±1 non-hub=36.6±0.5 | 0.7 |
| Average coding read depths of hub and non-hub targets | hub=37±0.2 non-hub=37.1±0.2 | 0.8 |
